# Molecular characterization of Pseudomyxoma peritonei with single-cell and bulk RNA sequencing

**DOI:** 10.1038/s41597-025-04561-4

**Published:** 2025-02-05

**Authors:** Ye Jin Ha, Seong-Hwan Park, Seon-Kyu Kim, Ka Hee Tak, Jeong-Hwan Kim, Chan Wook Kim, Yong Sik Yoon, Seon-Young Kim, Jong Lyul Lee

**Affiliations:** 1 Asan Institute for Life Sciences, Asan Medical Center, Seoul, 05505 Korea; 2 Aging Convergence Research Center, Korea Research Institute of Bioscience and Biotechnology (KRIBB), Daejeon, 34141 Korea; 3 Department of Bioscience, University of Science and Technology, Daejeon, 34113 Korea; 4 Personalized Genomic Medicine Research Center, KRIBB, Daejeon, 34141 Korea; 5 Division of Colon and Rectal Surgery, Department of surgery, University of Ulsan College of Medicine, Asan Medical Center, Seoul, 05505 Korea; 6 Korea Bioinformation Center, KRIBB, Daejeon, 34141 Korea

**Keywords:** Diagnostic markers, Cancer genomics

## Abstract

Pseudomyxoma peritonei (PMP), a rare condition characterized by mucinous ascites in the peritoneal cavity, often leads to a poor prognosis. However, omics profiling of this disease remains significantly underexplored. Here, we present single-cell transcriptomic profiling of five PMP cases to identify cell type-specific gene features associated with PMP pathogenesis. Additionally, we provide bulk RNA-seq datasets from two independent cohorts: 19 fresh frozen tissue samples (12 PMPs) and 34 formalin-fixed paraffin-embedded (FFPE) samples (25 PMPs). We also offer protein expression data from a tissue microarray (TMA) analysis of 90 samples (45 PMPs). Our single-cell and bulk transcriptomic profiles, along with TMA verifications, reveal the cellular diversity of PMP, highlighting the coexistence of epithelial and mesenchymal characteristics within PMP cells. These datasets enhance our understanding of PMP pathogenesis and provide a valuable resource for uncovering the intricate molecular landscape of PMP, with the potential to improve clinical utility through further research.

## Background & Summary

Pseudomyxoma peritonei (PMP) is a clinical condition characterized by the presence of gross mucinous or gelatinous ascites with mucinous implants on the peritoneal area^1^. PMP can be caused by mucinous neoplasms of various organs: appendix, ovary, colon, pancreas, gallbladder, and small intestine^2–4^. It is most commonly associated with peritoneal spread of a mucinous appendiceal neoplasm^3^. Although PMP is a rare condition with approximately 1 to 2 cases per 1 million persons per year in Europe and classified as a low-grade malignancy^5^, its indolent behavior often results in delayed diagnosis, thus leading to high mortality rates^3^.

It is believed that the pathogenesis of PMP involves the implantation of epithelial cells in the peritoneum after perforation on a low-grade appendiceal neoplasm, which produces mucin^6^. These mucin-producing cells can spread throughout the abdominal cavity, from the diaphragm to the pelvis^5^. While PMP is classified as a “borderline malignancy,” tumors confined to the appendix have a good prognosis; however, extra-appendiceal neoplasms have a poor survival outcome^6^. Recently, the terminology, diagnostic definition, and classification of PMP in 2012 were agreed upon by international experts who attended the World Congress of the Peritoneal Surface Oncology Group Internal^7,8^. Although cytoreductive surgery with hyperthermic intraperitoneal chemotherapy is the standard treatment for PMP and peritoneal seeding of colorectal cancer^8^, the survival rates vary depending on the classification, from a 10-year survival rate of 68% to as low as 3%^9^. The low incidence of PMP and its varied prognosis make it difficult to conduct large clinical and genomic studies. Genomic profiling is a promising strategy for early detection and for guiding additional treatment; however, its application in PMP is relatively underexplored. Despite being a rare tumor with low cellularity, genetic analysis of PMP is crucial for identifying therapy targets for treatments and improving early detection owing to its diverse but poor prognosis.

This study aims to advance knowledge on PMP by providing large-scale molecular profiles to identify key molecular markers. We utilized integrative techniques for single-cell and bulk transcriptomic profiling to explore the transcriptomic features of PMP. Our technical validation revealed active expressions of both epithelial and mesenchymal genes, highlighting the disease’s complex nature. Additionally, we verified practical protein markers encoded by hub genes associated with the epithelial and mesenchymal characteristics of PMP. The data generated from this study provide valuable molecular and clinical potential for further research.

## Methods

### Patients and tissue samples

Our study involved multiple cohorts from Asan Medical Center (Seoul, Korea) between 2005 and 2022 using peritoneal tissue samples. The first cohort, with five PMP patients, facilitated single-cell transcriptomic profiling to uncover cell type-specific gene signatures in PMP pathogenesis. Five fresh tissues were collected during surgery. The second cohort included 19 fresh frozen samples (12 PMPs, 7 healthy samples) for bulk RNA sequencing (RNA-seq). Written informed consent was obtained from the first and second cohorts for tissue samples and data sharing for this study. Additionally, a third cohort of 34 formalin-fixed paraffin-embedded (FFPE) samples (25 PMPs, 9 healthy samples) served for RNA-seq data validation. For protein expression verification through tissue microarray (TMA) analysis, a fourth cohort of 90 samples (45 PMPs, 45 healthy samples) was utilized. The requirement for informed consent was waived because of the retrospective nature for the third and fourth cohorts. The study protocol was strictly approved by the Institutional Review Board of Asan Medical Center (registration number: 2017–0681), and the study was conducted in accordance with the Declaration of Helsinki. Table 1 details the clinical characteristics of these patients. Patients were enrolled if their tumors were pathologically diagnosed as PMP and intraoperative findings revealed mucinous peritoneal implants. Patients were excluded if their tumors were diagnosed as appendiceal mucinous neoplasms without PMP, adenocarcinoma, or peritoneal metastases from gastrointestinal, gynecologic, or other sites, or if they had other concurrent malignancies. Patients were also excluded if re-evaluation of histologic findings was unavailable due to loss of key slides. H&E staining was analyzed by a pathologist to determine the percentage of tumor cellularity of the samples for RNA-seq and summarized in Table S1. Images of H&E staining of PMP samples for single-cell RNA sequencing (scRNA-seq) were in Fig. S1.

**Table 1:** Clinical characteristics of the enrolled PMP patients.

	Discovery cohort			TMA cohort	*P* value*
	1st cohort (n = 5)	2nd cohort (n = 12)	3rd cohort (n = 25)	4th cohort (n = 45)	
Age at operation, year	61 ± 6	62 ± 13	56.88 ± 12	58.81 ± 12	0.67
Sex					0.84
Male	2 (40)	3 (25)	10 (40)	16 (36)	
Female	3 (60)	9 (75)	15 (60)	29 (64)	
Operation time, min	459 ± 267	453 ± 199	302 ± 215	300 ± 191	0.06
Hospital stay, day	15 ± 6	14 ± 7	13 ± 6	13 ± 8	0.96
BMI	20.76 ± 2.82	24.75 ± 1.99	24.07 ± 2.94	24.15 ± 2.77	0.05
Follow-up period, month	21 ± 7	45 ± 33	60 ± 45	51 ± 41	0.24
PreCEA					0.35
<5	2 (40)	4 (33)	4 (16)	6 (13)	
>5	3 (60)	8 (67)	12 (48)	26 (58)	
unknown	0 (0)	0 (0)	9 (36)	13 (29)	
PCI, 0-39	30 ± 8	23 ± 7	22 ± 10	21 ± 10	0.33
LVI					0.70
Yes	0 (0)	0 (0)	2 (8)	4 (9)	
No	4 (80)	8 (67)	15 (60)	33 (73)	
unknown	1 (20)	4 (33)	8 (32)	8 (18)	
PNI					0.12
Yes	0 (0)	0 (0)	1 (4)	1 (2)	
No	4 (80)	4 (33)	14 (56)	34 (76)	
unknown	1 (20)	8 (67)	10 (40)	10 (22)	
Histological grading					0.71
Low	4 (80)	12 (100)	19 (76)	35 (78)	
High or SRC	1 (20)	0 (0)	6 (24)	10 (22)	
Recurrence					0.06
Yes	1 (20)	9 (75)	19 (76)	34 (76)	
No	4 (80)	3 (25)	6 (24)	11 (24)	
Postoperative complication					0.81
Yes	4 (80)	9 (75)	16 (64)	29 (64)	
No	1 (20)	3 (25)	9 (36)	16 (36)	
Comorbidity					0.80
Yes	3 (60)	7 (58)	11 (44)	21 (47)	
No	2 (40)	5 (42)	14 (56)	24 (53)	
IP chemo. or HIPEC					0.06
Yes	4 (80)	9 (75)	10 (40)	18 (40)	
No	1 (20)	3 (25)	15 (60)	27 (60)	
CCR					0.23
0 or 1	4 (80)	8 (67)	10 (40)	21 (47)	
2 or 3	1 (20)	4 (33)	15 (60)	24 (53)	

Values are presented as mean ± SD or case number (%).

*Comparison by ANOVA or χ^2^ tests.

Abbreviations: PMP, psuedomyxoma peritonei; BMI, body mass index; LVI, lymphovascular invasion; PNI, perineural invasion; SRC, signer-ring cell; CEA, carcinoembryonic antigen; PreCEA, preoperation CEA; IP chemo., intraperitoneal chemotherapy; HIPEC, hyperthermic intraperitoneal chemotherapy; CCR, completeness of cytoreduction (0: no macroscopic, 1: < 2.5 mm, 2: 2.5mm–2.5 cm, 3: > 2.5 cm); PCI, peritoneal carcinoma index.

### Single-cell isolation

Tissue samples from five patients were processed using the Human Tumor Dissociation Kit (Miltenyi Biotec, Germany) for dissociation into cell suspensions. These suspensions were then sieved through a 40-μm cell strainer (pluriSelect, Germany) and washed with phosphate buffered saline containing 0.04% bovine serum albumin. This was followed by red blood cell removal using a 10-minute treatment with ammonium–chloride–potassium Lysing Buffer (Thermo Fisher Scientific, MA, USA). Live cells were subsequently isolated using the Dead Cell Removal Kit (Miltenyi Biotec) and quantified with a Countess II Automated Cell Counter (Thermo Fisher Scientific) after 0.2% trypan blue staining.

### Transcriptomic profiling based on scRNA-seq

The protoplast suspension was processed using Chromium microfluidic chips with 30 v3 chemistry and a 10 × Chromium Controller (10 × Genomics, CA, USA) for barcoding. RNA from these barcoded cells underwent reverse transcription, and sequencing libraries were created with the Chromium Single Cell 30 v3 Reagent Kit (10 × Genomics), followed by sequencing on the NovaSeq 6000 (Illumina, CA, USA). Data processing, including quality control and gene counting, was conducted using Cell Ranger Single Cell Software Suite ver. 6.0 (10 × Genomics). We note that for the integration analysis of scRNA-seq data from PMP and healthy sample, we obtained single-cell transcriptome data of healthy sample from the supporting information of a previous study^10^.

For scRNA-seq data analysis, we utilized the *Seurat* package in R software, integrating data from multiple samples and segregating cells into clusters using the Louvain algorithm. Cell type for each cluster was identified using gene markers from the *Seurat* package’s *FindMarkers* function, validated through DAVID software. We identified variable gene sets across cell clusters by calculating the coefficient of variation in counts per million mapped reads (CPM) for each cell. Cell similarity was evaluated through principal component analysis of these variable genes, and clustering results were visualized using uniform manifold approximation and projection based on these components. For pseudo-bulking from scRNA-seq data, we used Seurat’s ‘AggregateExpression’ method and generated a single gene expression profile for each sample type.

### RNA extraction, RNA-seq experiments, and data processing

Total RNA from fresh frozen tissue samples was extracted using TRIzol reagent (Invitrogen, CA, USA) and stored at −80 °C. RNA quality was assessed using RNA Integrity Number (RIN) and DV200 metric, determined by RNA 6000 Nano Kit and Agilent 2100 Bioanalyzer (Agilent Technologies, CA, USA), selecting samples with RIN ≥ 8 for analysis.

For FFPE samples, sections from FFPE block were prepared followed by hematoxylin-eosin staining of one slide. Tumor tissues were microdissected from subsequent unstained sections and used for RNA preparation. Total RNA was extracted using RNeasy FFPE Kit (Qiagen, CA, USA) and stored at −80 °C, with integrity checked by Agilent 2100 Bioanalyzer, requiring RIN ≥ 7.

Sequencing libraries were prepared from fresh frozen samples using TruSeq Stranded mRNA LT Sample Prep Kit, and for FFPE samples using TruSeq RNA Access Library Prep Kit (both Illumina). mRNA purification from total RNA, cDNA conversion, adapter ligation, and PCR amplification were performed, followed by paired-end sequencing (2 × 100 bp) on the NovaSeq platform (Illumina). We used FASTQC (ver. 0.11.9) to perform quality control checks on raw sequencing reads, ensuring data reliability and integrity. Reference genome data (*Homo sapiens*, GRCh38) from NCBI Genome database were used for genome indexing and read mapping using STAR software (ver. 2.5.4b).

### Transcriptomic profiling of bulk RNA-seq data

To analyze mRNA expression across tissue samples, we applied hierarchical clustering using the centered correlation coefficient for similarity and centroid linkage. Gene expression levels were estimated with CPM values and normalized via the quantile method, log2-transformed, and median-centered.

To identify differentially expressed genes, we used the edgeR package^11^, employing a negative binomial model and Cox–Reid profile-adjusted likelihood for dispersion estimation. A gene filtering strategy was applied to remove non-expressed genes, retaining those with CPM > 1 in at least three samples. This process resulted in 16,511 genes being included in the downstream analysis. Differentially expressed genes were identified using a generalized linear model (GLM) likelihood ratio test, fitting negative binomial GLMs with Cox–Reid dispersion. Statistical significance was set at *P < *0.001, with a minimum fold difference of ≥1.5 between sample groups. To compare expression levels between fresh-frozen and FFPE samples while minimizing batch effects, we applied ComBat-Seq^12^, a batch correction method based on a negative binomial regression model, designed to retain the integer nature of count data. For estimating cell type proportions in bulk RNA-seq data, we used EPIC software (Epic systems, WI, USA)^13^ and CIBERSORTx^14^, referencing gene markers from 10 cell clusters identified in single-cell transcriptomic profiling.

## Data Records

Single cell RNA-seq dataset for PMP is available the National Center for Biotechnology Information (NCBI) Gene Expression Omnibus (GEO; https://www.ncbi.nlm.nih.gov/geo) under accession number GSE228377^15^. To facilitate the reproduction of single-cell transcriptomic profiling, RDS (a file format to save R objects) files for the five PMP scRNA-seq samples are included in the same GEO accession. Additionally, bulk RNA-seq datasets are available in NCBI GEO repository under accession numbers GSE228375^16^ and GSE228376^17^. The TMA raw data and their corresponding clinical data are available in Supplementary Tables S2 and S3, respectively.

### Data Format and Structure

- FASTQ Files: Contain raw sequencing reads from single-cell and bulk RNA-seq experiments.
- TMA Data: Protein expression data for E-cadherin, FGFR2, TGF-α, and PI3K-γ are available in Supplementary Table S2.

## Technical Validation

### Single-cell transcriptome data analysis in PMP

**Fig. 1:**
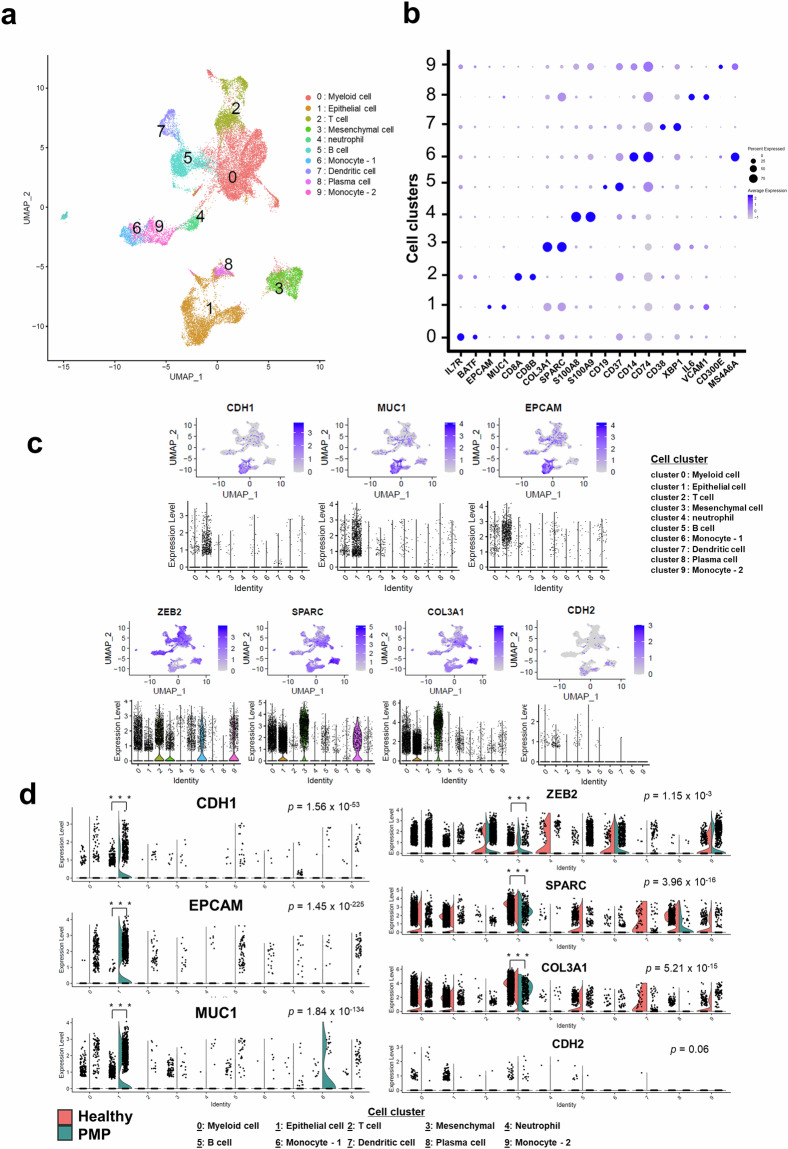
Diverse cell types identified via integrative single-cell transcriptomic analysis in pseudomyxoma peritonei (PMP) and healthy. (**a**) The uniform manifold approximation and projection plot shows the distinct cell types identified in all samples. (**b**) The dot map shows the expression levels of specific markers in each cell type. (**c**) The feature and violin plots display the expression patterns of epithelial-specific (upper panel) and mesenchymal-specific (lower panel) markers in the single-cell transcriptomic data of PMP and healthy sample (**d**) The feature plots present scatter plots depicting the distribution of epithelial-specific (left panel) and mesenchymal-specific (right panel) markers, highlighting the distinctions between PMP and healthy. *P*-values were calculated using the Wilcoxon rank-sum test with Bonferroni adjustment; ***P < 0.001.

Quality assessments of the scRNA-seq data obtained from each PMP sample showed that the data met the quality control standards of the 10 × genomics platform (Supplementary Table S4). When estimating the optimal dimensionality of the integrated scRNA-seq data from PMP and healthy sample^10^, we identified the six dimension as the elbow point (Supplementary Fig. S2a), indicating that six principal components (PCs) were sufficient to determine optimal cell communities. Then, we used this dimensionality data to identify optimal cell clusters based on the Silhouette index^18^ (Supplementary Fig. S2b, c), showing that 10 cell communities were optimal (Fig. 1a). To further evaluate the sufficiency of six PCs, we repeated the analysis with an expanded set of 16 PCs and again identified 10 cell communities as optimal. Adjusted Rand Index (ARI) analysis also confirmed that 10 clusters provided the highest stability across both PC selections, further validating the robustness of our clustering results regardless of the number of PCs used (Supplementary Fig. S2b). Supplementary Table S5 presents a list of genes significantly expressed in each cell cluster of PMP and healthy sample.

Before delving into the characterization of the cell lineages within the 10 clusters, we investigated the cell compositions of individual samples and presented them in sample groups of PMP and healthy sample (Supplementary Fig. S3a, b). We observed some differences in cell composition between the PMP and healthy sample groups, which may be attributed to tissue differences (Supplementary Fig. S3b). Despite the varying proportions of cells from the two tissues, there was substantial overlap of cells across the various clusters (Supplementary Fig. S3c, d). This suggests that the single-cell gene expression data from individual samples were effectively normalized, enabling a comprehensive analysis across different samples.

To characterize the 10 distinct cell clusters, we examined enriched functions within each cluster (Supplementary Fig. S4). Our analysis revealed the presence of diverse immunological cell groups, such as myeloid cells, T cells, neutrophil, B cells, monocytes, dendritic cells, and plasma cells. Furthermore, we identified exclusive communities of epithelial (cell cluster 1) and mesenchymal (cell cluster 3) cells (Fig. 1a). The expression levels of specific markers within each cell type indicated that these markers were generally well represented in their corresponding cell groups compared with others (Fig. 1b). Supplementary Table S6 presents a comprehensive list of gene markers specifically associated with the 10 distinct cell clusters. To further validate and confirm the characteristics of the active epithelial and mesenchymal cell communities, we identified representative genes for each and examined their expression levels across the different cell clusters. The uniform manifold approximation and projection plots in Fig. 1c show the presence of well-known markers for epithelial cells (upper panel) and mesenchymal cells (lower panel) across all samples. Most markers for epithelial cells were exclusively expressed in the epithelial cell group (cell cluster 1), whereas markers for mesenchymal cells exhibited higher expressions in the mesenchymal cell group (cell cluster 3) and relatively increased expressions across other cell clusters (Fig. 1c). Although *CDH2*, a representative mesenchymal marker, exhibited high expression in the mesenchymal cell group, there was no statistical significance (Supplementary Fig. S5). Overall, our analysis demonstrates the molecular heterogeneity of integrated cells from PMP and healthy samples, highlighting the presence of distinct epithelial and mesenchymal cell groups simultaneously.

### Characteristics of cell composition of PMP

In PMP samples, epithelial markers were significantly elevated compared to healthy samples, particularly within the epithelial cell cluster (left panel in Fig. 1d). Conversely, mesenchymal markers showed higher expression in the mesenchymal cell cluster of PMP, while being evenly expressed across all cell communities in healthy samples (right panel in Fig. 1d). Supplementary analyses confirmed these patterns, with epithelial and mesenchymal markers exhibiting distinct distributions in PMP and healthy samples (Supplementary Figs. S6–S9). These findings highlight the molecular heterogeneity of PMP, characterized by distinct epithelial and mesenchymal cell populations contributing to the aggressive nature of the disease.

To gain further insights into the subtypes of epithelial and mesenchymal cells from both healthy and PMP samples, we conducted a subset analysis of these groups. In epithelial cells, we found distinct sub-cell groups, with healthy samples showing mesothelial and endothelial markers, while PMP samples exhibited strong mucin-associated gene expression and markers of gastrointestinal mucinous carcinoma (Supplementary Fig. S10). Similarly, mesenchymal cell analysis revealed some mesothelial characteristics in both healthy and PMP samples, but with low expression of mesothelioma markers, suggesting a low likelihood of association with mesothelioma (Supplementary Fig. S11).

### Validation of the molecular heterogeneity of PMP through bulk RNA-seq profiling

**Fig. 2:**
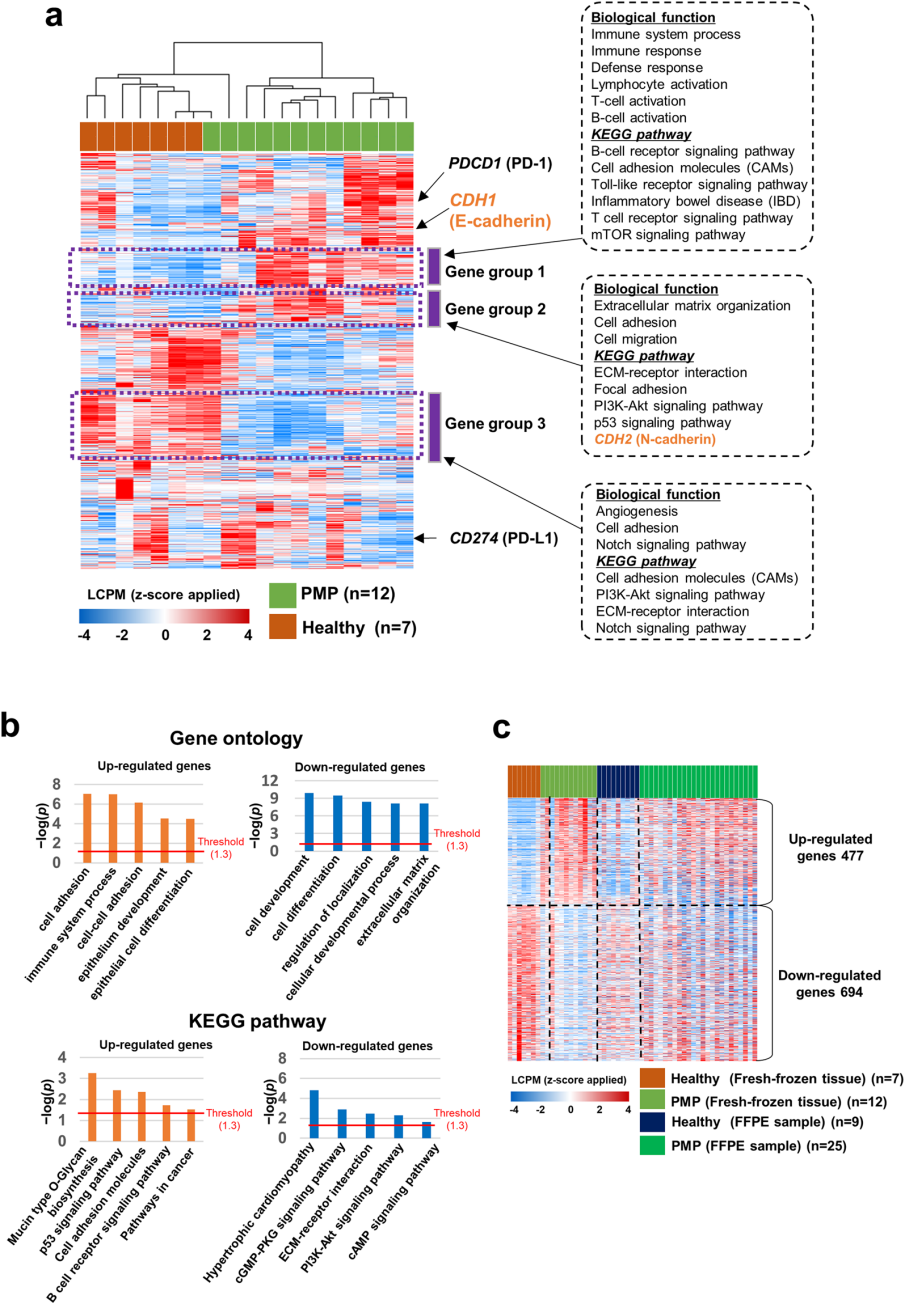
Comparison of the expressions between pseudomyxoma peritonei (PMP) and healthy control tissues. (**a**) Unsupervised hierarchical cluster analysis of expressions in PMP and healthy control tissues. In this analysis, 4,737 genes showing expression changes with standard deviation of 0.8 or more were used. (**b**) Function enrichment analyses of 1,171 differentially expressed genes between PMP and healthy control tissues. The analysis was conducted using the Gene Ontology biological process and KEGG pathway. (**c**) Heatmap depicting the expression patterns of 1,171 genes in fresh frozen and formalin-fixed paraffin-embedded samples. LCPM, log-transformed counts per million.

To validate the molecular heterogeneity of PMP, we conducted bulk RNA-seq analysis on fresh frozen tissues (second cohort, n = 19), revealing distinct gene expression patterns that separated PMP from healthy samples (Fig. 2a). Differential expression analysis identified 1,171 genes, with significant enrichment in pathways related to cell adhesion, immune response, and epithelial development, consistent with the scRNA-seq findings (Fig. 2b, Supplementary Fig. S12). Batch-corrected analysis of FFPE samples (third cohort, n = 34) using ComBat-Seq confirmed moderately similar expression changes between PMP and healthy samples, reinforcing the robustness of our findings (Fig. 2c, Supplementary Fig. S13). Pseudo-bulk analysis from scRNA-seq further supported these observations, highlighting genes associated with proliferation and immune processes (Supplementary Fig. S14). Cell fraction analysis using EPIC^13^ and CIBERSORTx^14^ validated significant differences in epithelial and mesenchymal populations between PMP and healthy tissues (Supplementary Fig. S15), aligning with scRNA-seq data (Supplementary Fig. S6). These findings confirm that PMP exhibits distinct epithelial and mesenchymal characteristics at both single-cell and bulk transcriptomic levels.

## Usage Notes

### Reproducibility

All methods used for data generation and analysis are described in detail within the Methods section. Key parameters and software versions are provided to ensure reproducibility. The study includes single-cell transcriptomic profiling, bulk RNA-seq, and TMA data.

### Potential Limitations

Users should be aware of the following limitations:

- Sample Size: Compared to more common diseases such as cancer, the relatively small sample size of PMP patients may limit the generalizability of the findings.
- Cell Line Models: The lack of a cell line model of PMP due to its heterogeneity makes *in vitro* validation challenging.

## Supplementary information

Supplementary figures

Supplementary tables

## Data Availability

All software and pipelines in this study were executed with parameters described in the Methods section. Default settings recommended by the software developers were used if specific parameters were not detailed. To facilitate reproducibility, the R code for both single-cell and bulk RNA-seq analyses is publicly available on GitHub at https://github.com/sunghwan1234290/PMP.git.
