# Supplementary material for: Molecular characterization of Pseudomyxoma peritonei with single-cell and bulk RNA sequencing
Source: Sci Data. 2025 Feb 5;12:213. doi: 10.1038/s41597-025-04561-4 (PMC11799422; doi:10.1038/s41597-025-04561-4)
Supplement: Supplementary file 1 — Supplementary figures [file 41597_2025_4561_MOESM1_ESM.pdf]

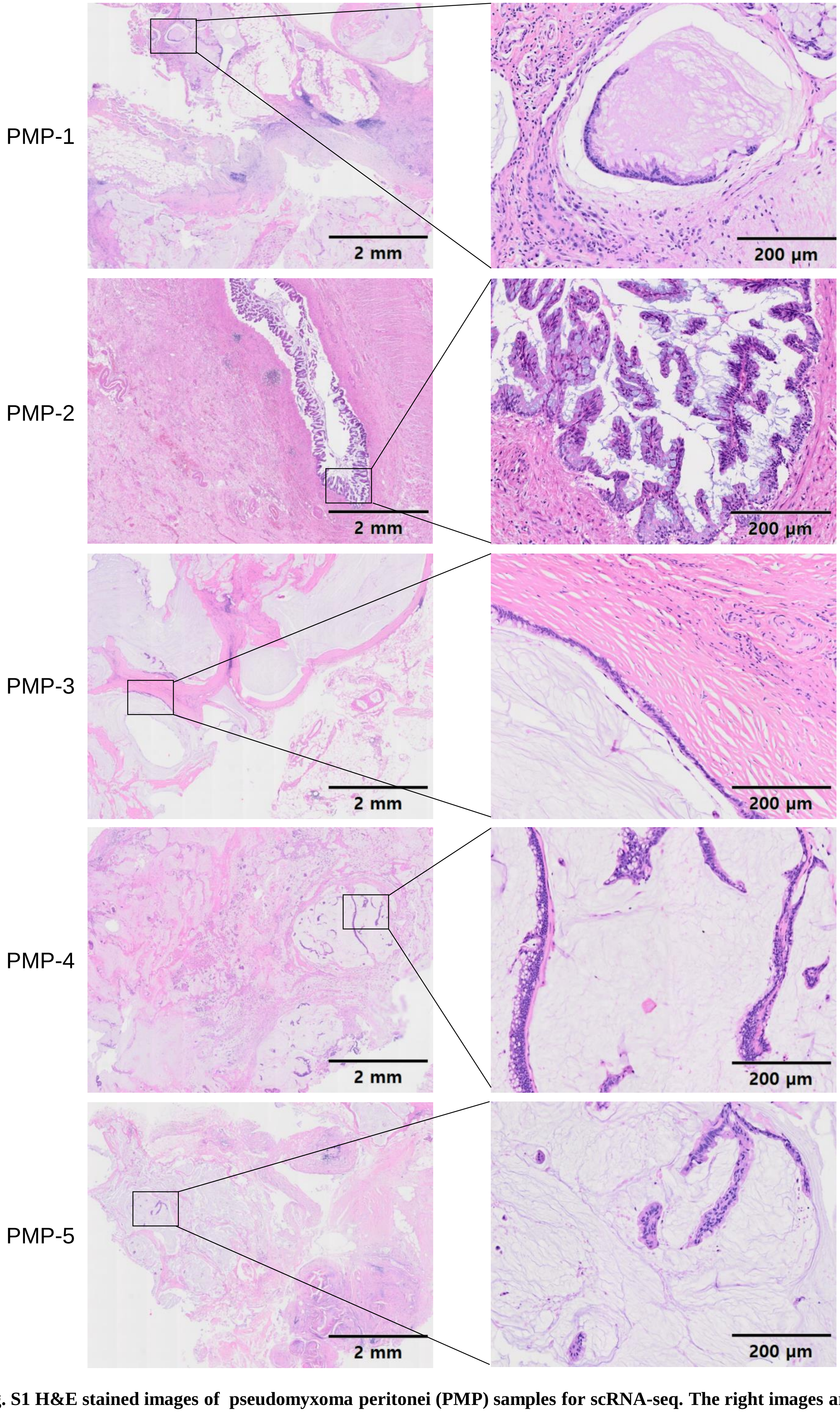

Fig. S1 H&E stained images of pseudomyxoma peritonei (PMP) samples for scRNA-seq. The right images are the tumor cell area in the left images. Left images: 10X, right images: 100X.

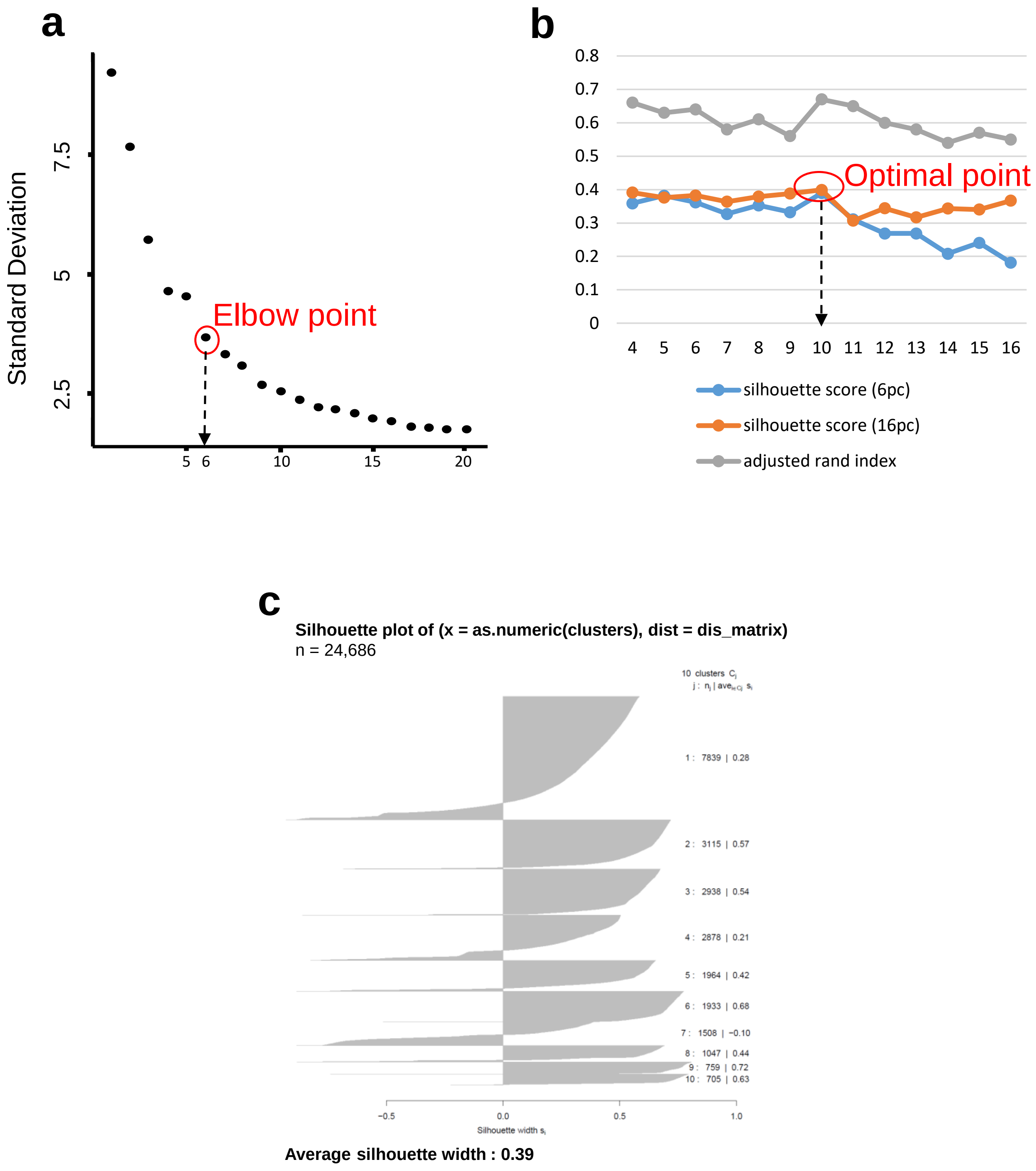

Fig. S2 Determination of optimal cell clusters in single-cell transcriptomic data of Pseudomyxoma peritonei and healthy sample.

(**a**) Identification of the elbow point to determine the appropriate number of principal components for analyzing cell communities. (**b**) Determination of the optimal number of cell clusters based on Silhouette score estimation, incorporating analyses using both six and sixteen principal components (PCs). In both cases, ten cell clusters were consistently identified as the optimal number. (**c**) Silhouette widths of all cells across clusters when K = 10, based on analysis using six PCs.

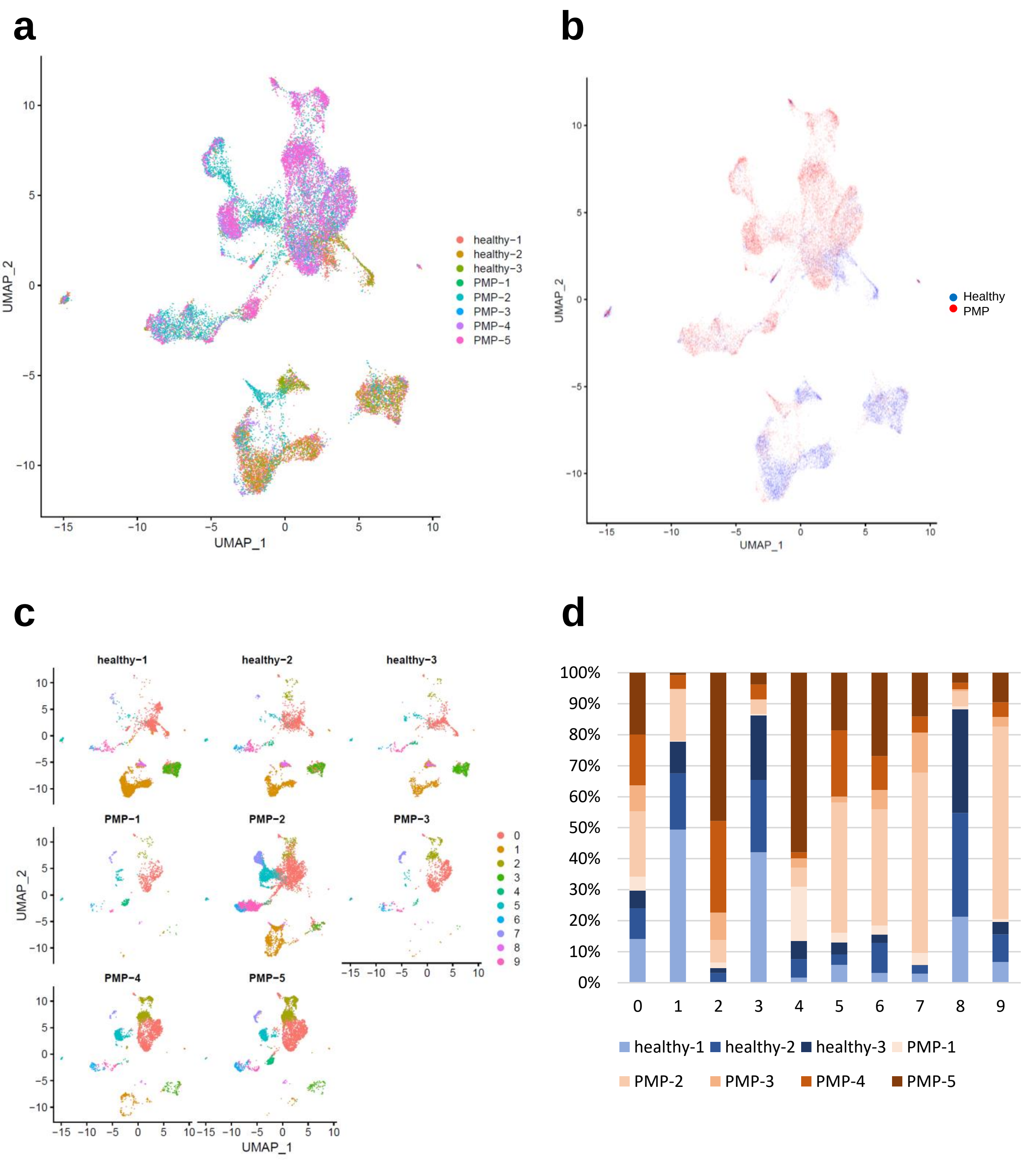

Fig. S3 Single-cell transcriptomic profiling of samples obtained from pseudomyxoma peritonei (PMP, n=5) and healthy sample (n=3).

(**a**) Uniform manifold approximation and projection (UMAP) plot showing the distribution of cells from individual samples with PMP or healthy. (**b**) UMAP plot illustrating the separation of cells from the two sample groups of PMP and healthy. (**c**) Multiple UMAP plots, each representing cells from individual samples with PMP or healthy, highlighting the overlap and distribution of cells across different samples**.** (**d**) Bar plot illustrating the proportion of cells from each sample within each cell cluster.

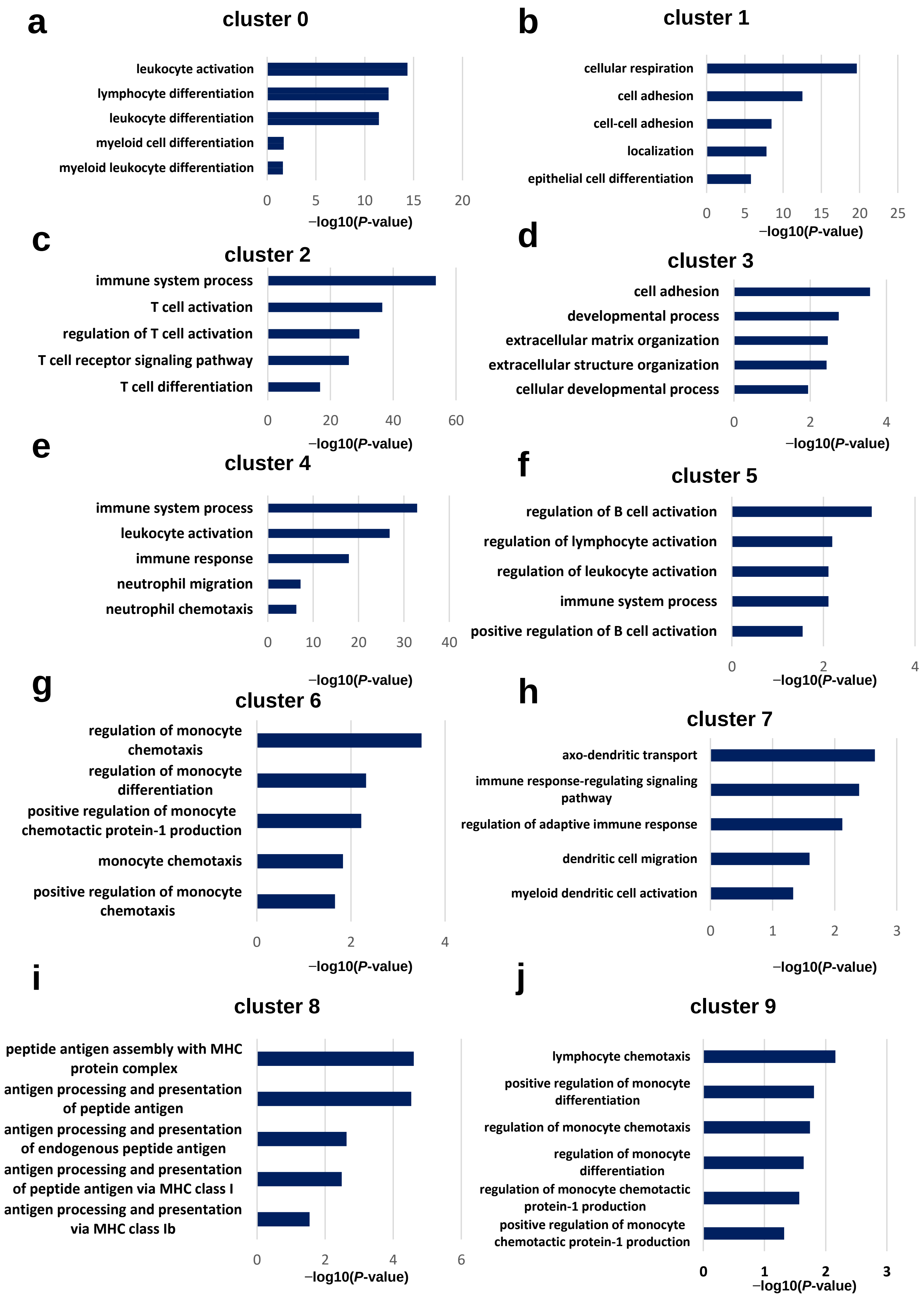

Fig. S4 Function enrichment tests of 10 cell clusters in pseudomyxoma peritonei (PMP) and healthy sample.

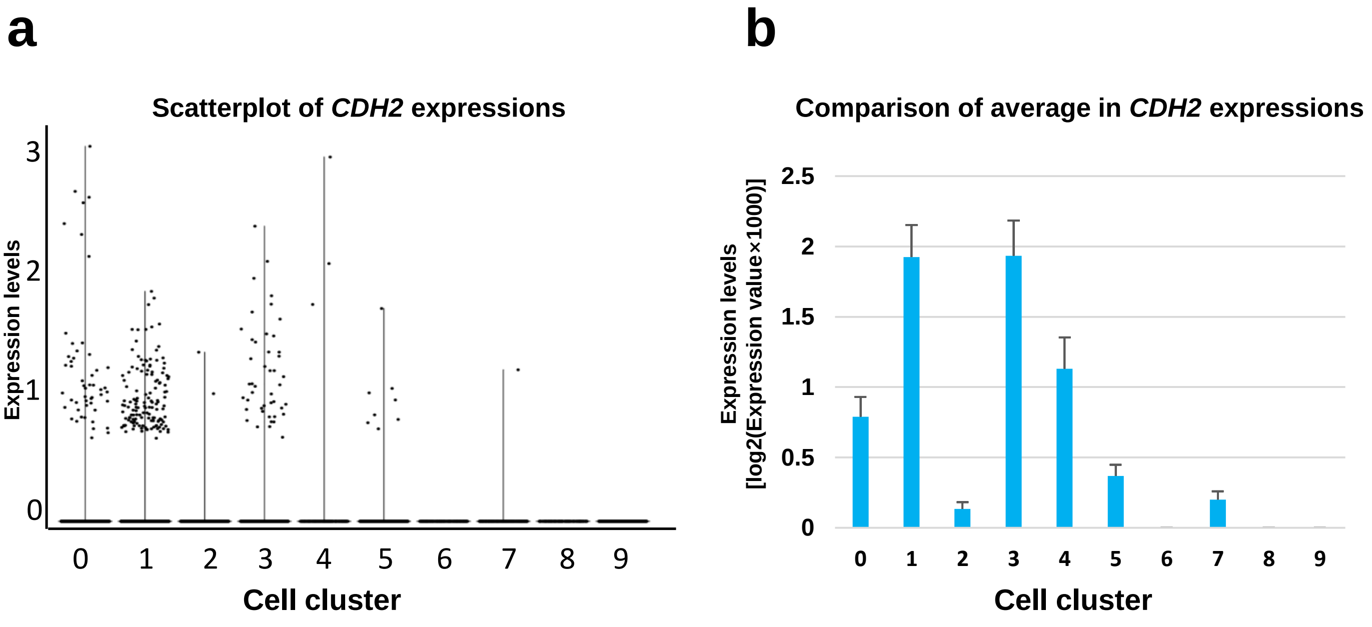

Cell cluster

0: Myeloid cell 1: Epithelial cell 2: T cell 3: Mesenchymal 4: Neutrophil
5: B cell 6: Monocyte 1 7: Dendritic cell 8: Plasma cell 9: Monocyte 2

Fig. S5 Comparison of *CDH2* expression levels across cell communities in pseudomyxoma peritonei (PMP) and healthy sample.

(**a**) Scatterplot of *CDH2* expression levels across 10 cell clusters. Raw expression levels of *CDH2* obtained using the *Seurat* tool are displayed. (**b**) Bar plot depicting *CDH2* expression levels across the 10 cell clusters. To emphasize differences between clusters, *CDH2* expression levels were scaled using log2 (raw expression values×1000) transformation. The upper and lower bars represent the standard deviation of expression values.

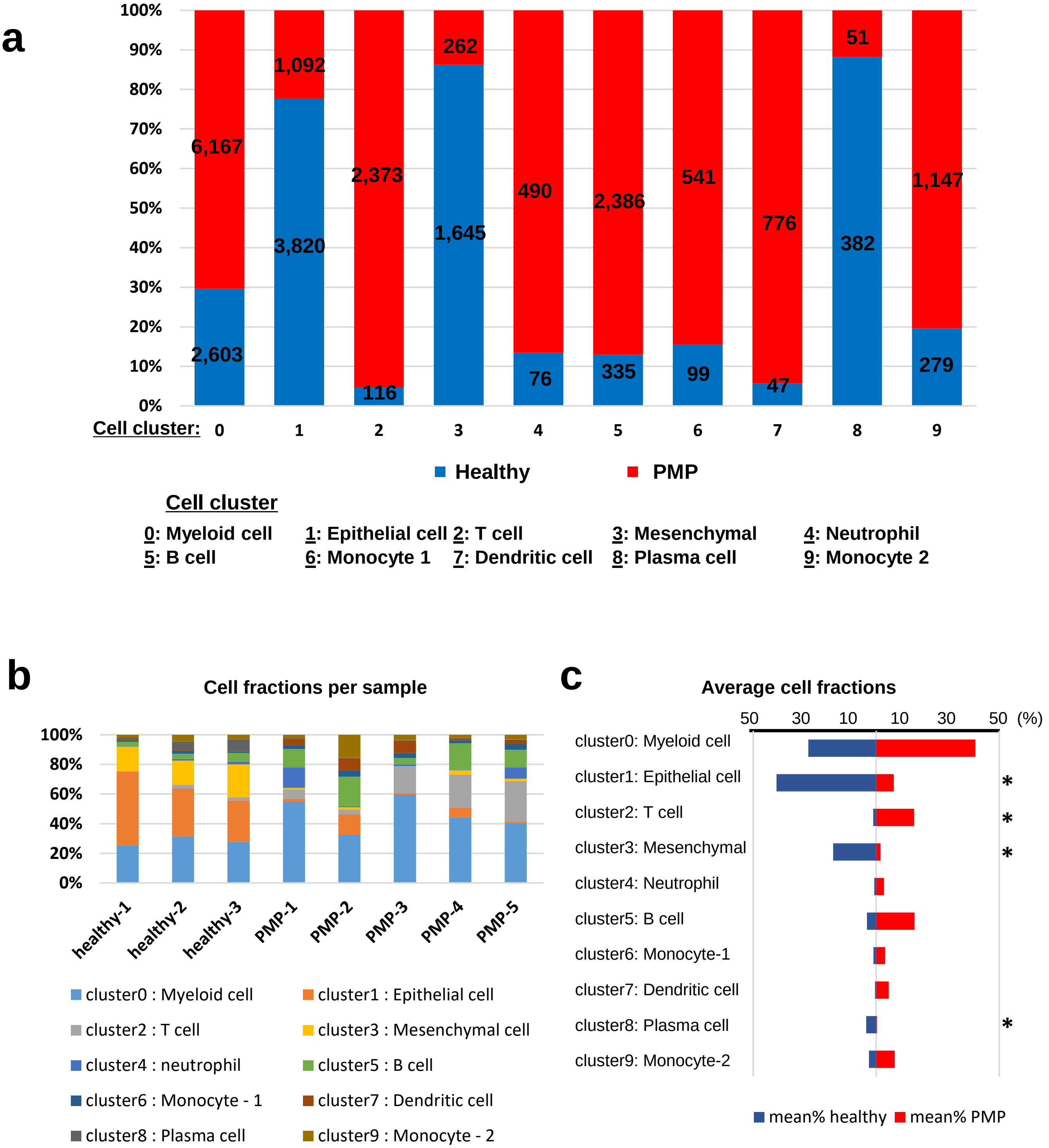

Fig. S6 Comparison of cell fractions between healthy sample and pseudomyxoma peritonei (PMP).

(**a**) Assessment of cell frequencies within clusters in healthy or PMP samples. (**b**) Evaluation of cell fractions within clusters for individual samples with healthy or PMP. (**c**) Comparative analysis of average cell fractions between samples with healthy and PMP. *, $P < 0.05$ by fisher's exact test.

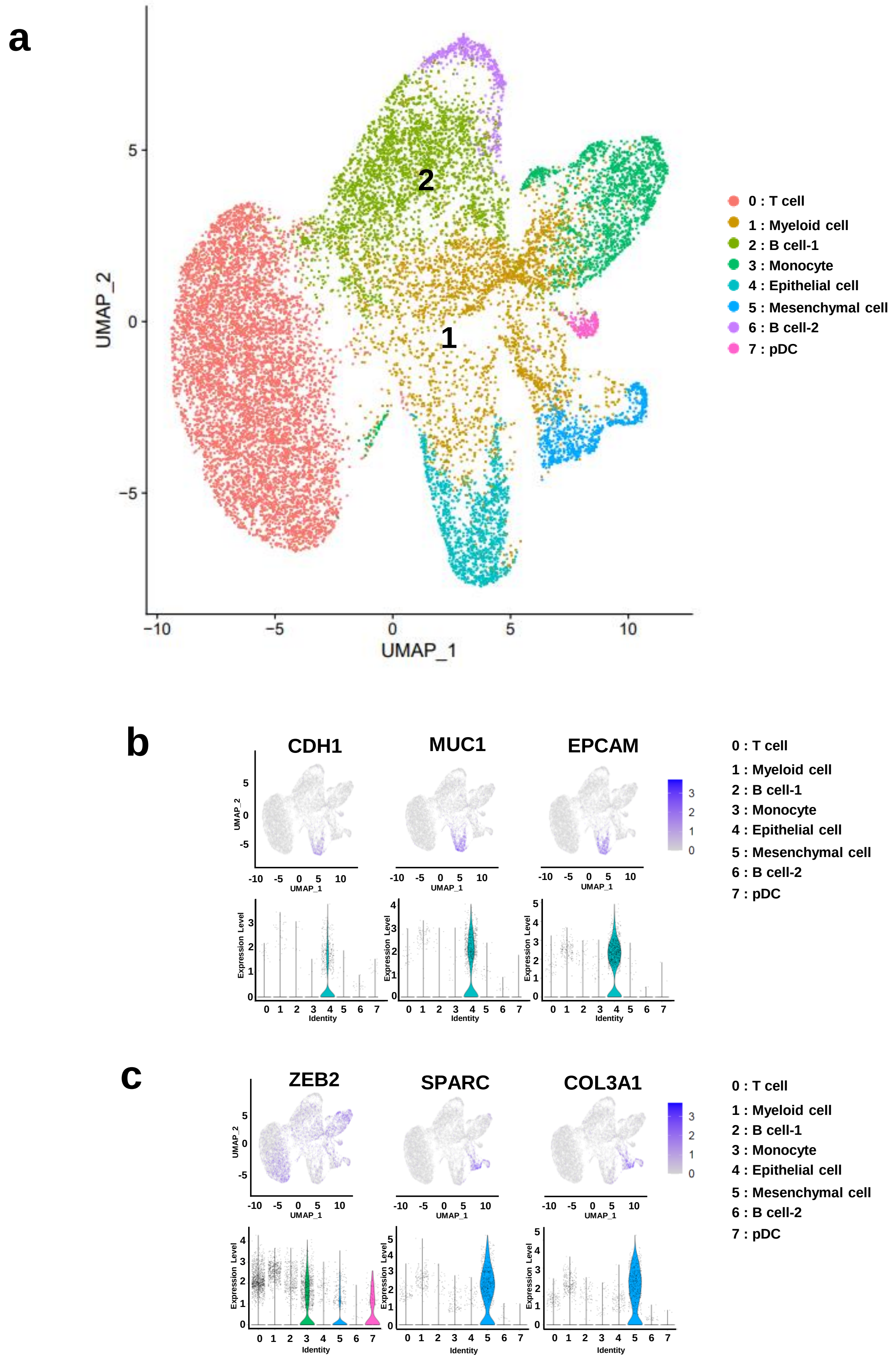

Fig. S7 Single-cell transcriptomic profiling of samples with pseudomyxoma peritonei (PMP).

(**a**) The uniform manifold approximation and projection plot shows the distinct cell types identified in PMP samples. (**b, c**) The feature and violin plots display the expression patterns of epithelial-specific (**b**) and mesenchymal-specific (**c**) markers in the single-cell transcriptomic data of PMP.

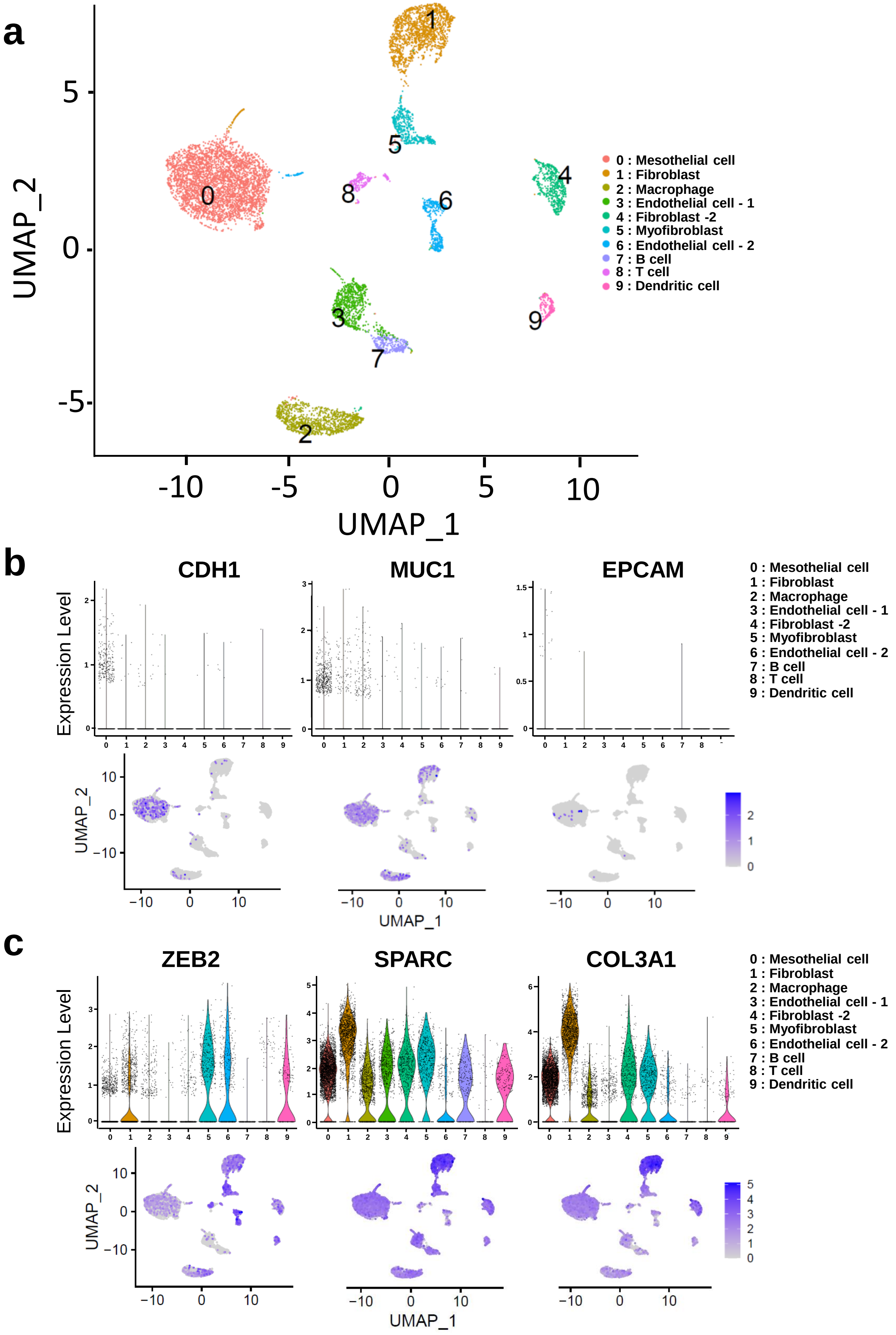

Fig. S8 Single-cell transcriptomic profiling of samples with healthy sample.

(**a**) The uniform manifold approximation and projection plot exhibiting the distinct cell groups in healthy samples. (**b**,**c**) The feature and violin plots display the expression patterns of epithelial-specific (**b**) and mesenchymal-specific (**c**) markers in the single-cell transcriptomic data of healthy samples.

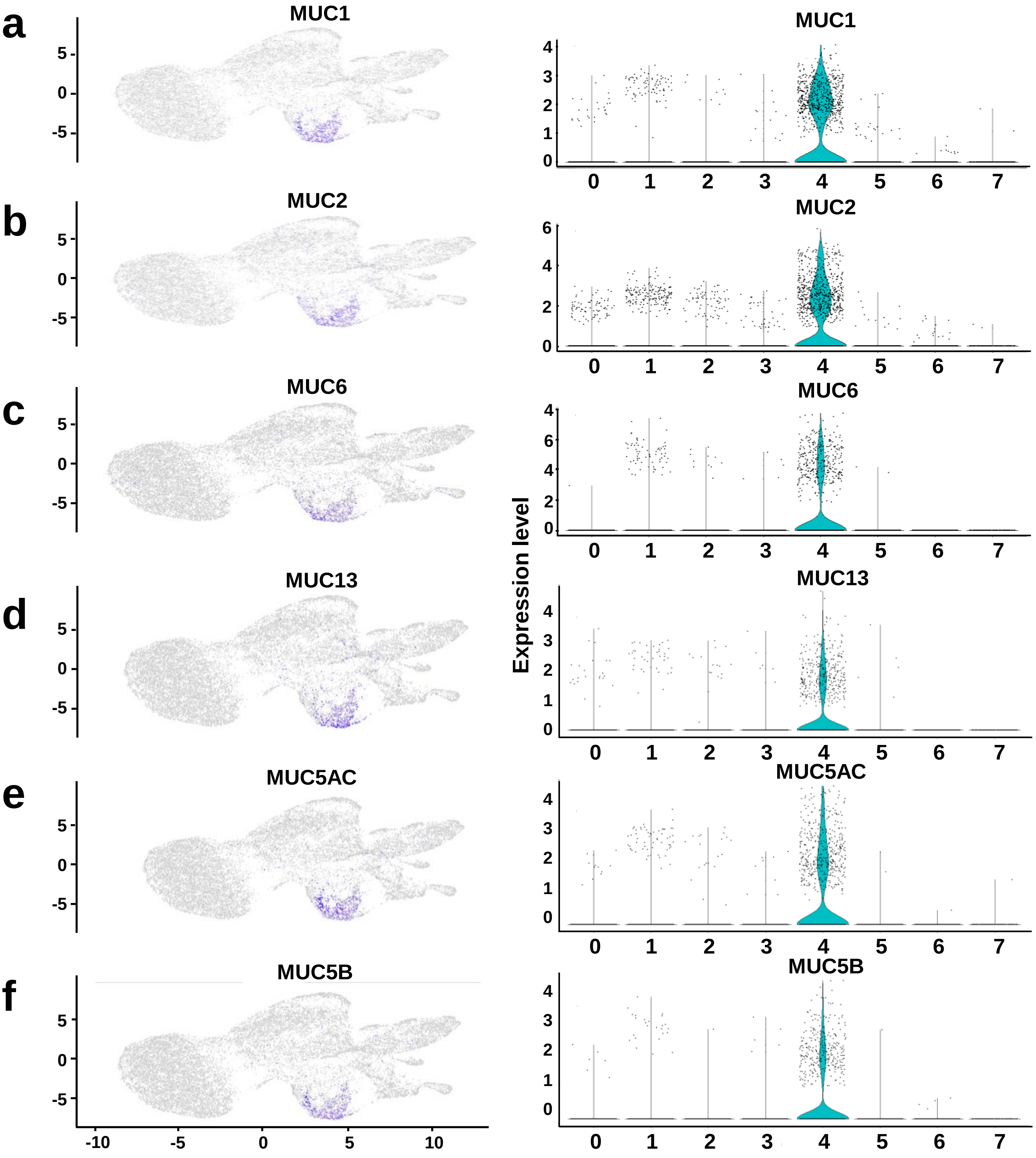

<u>Cell cluster</u>

0 : T cell
1 : Myeloid cell
2 : B cell-1
3 : Monocyte
4 : Epithelial cell
5 : Mesenchymal cell
6 : B cell-2
7 : pDC

Fig. S9 Expression profiles of mucinous genes in single-cell transcriptomic data of pseudomyxoma peritonei.

(**a-f**) Genes involved in mucin family were highly expressed in cluster 4 of the epithelial-associated cell group.

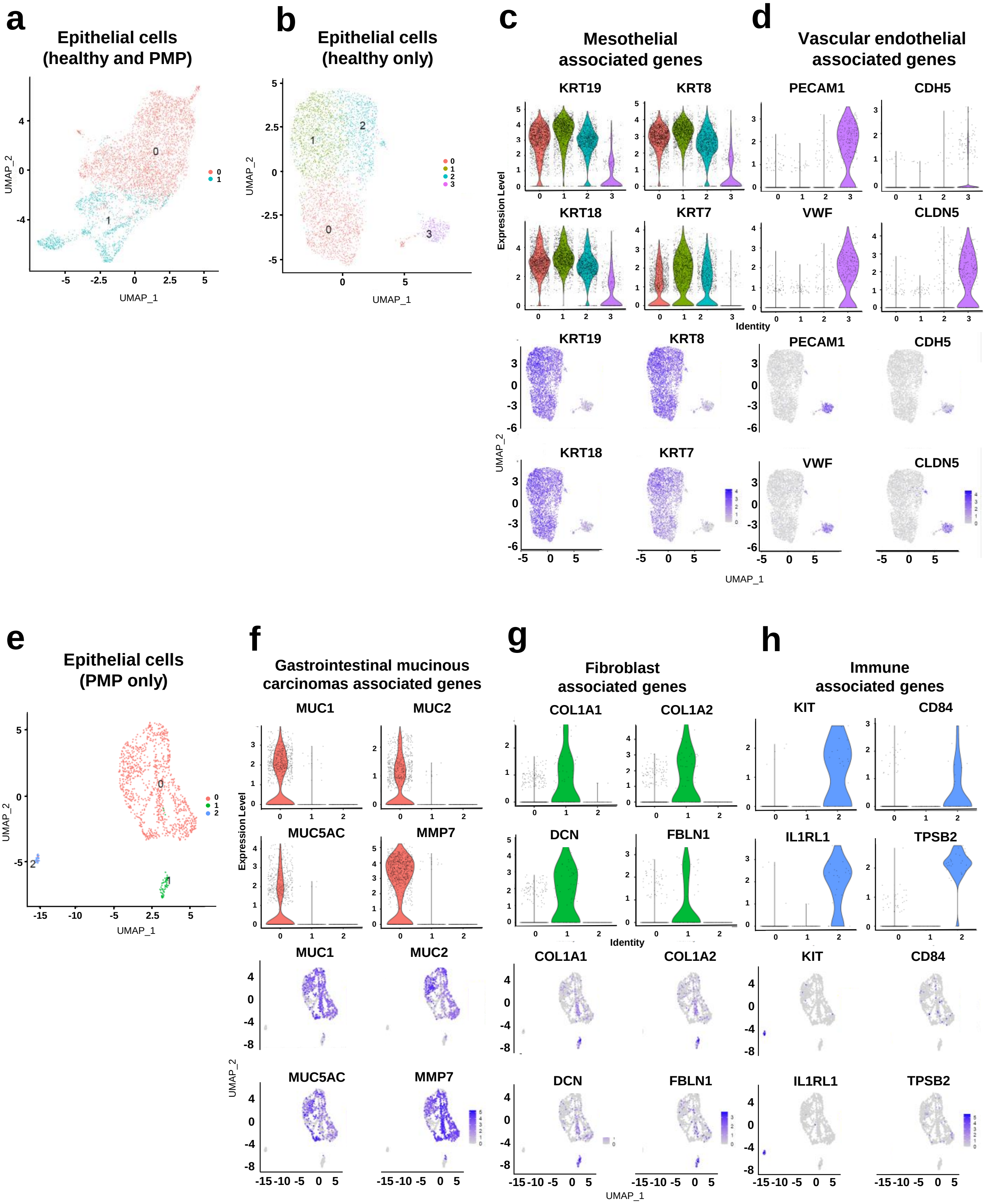

Fig. S10 Subset analysis of epithelial-characterized cells using uniform manifold approximation and projection (UMAP) plots.

(**a**) UMAP plot of epithelial cells, with colors representing Seurat-defined clusters. (**b**) UMAP plot of epithelial cells from healthy samples only, identifying four distinct Seurat clusters. (**c, d**) Expression patterns of (**c**) mesothelial (KRT family) and (**d**) endothelial markers, respectively, across Seurat clusters. (**e**) UMAP plot of epithelial cells from PMP samples only, identifying three distinct Seurat clusters. (**f, g, h**) Expression patterns of (**f**) gastrointestinal mucinous carcinoma markers, (**g**) fibroblast markers, and (**h**) immune cell markers, respectively.

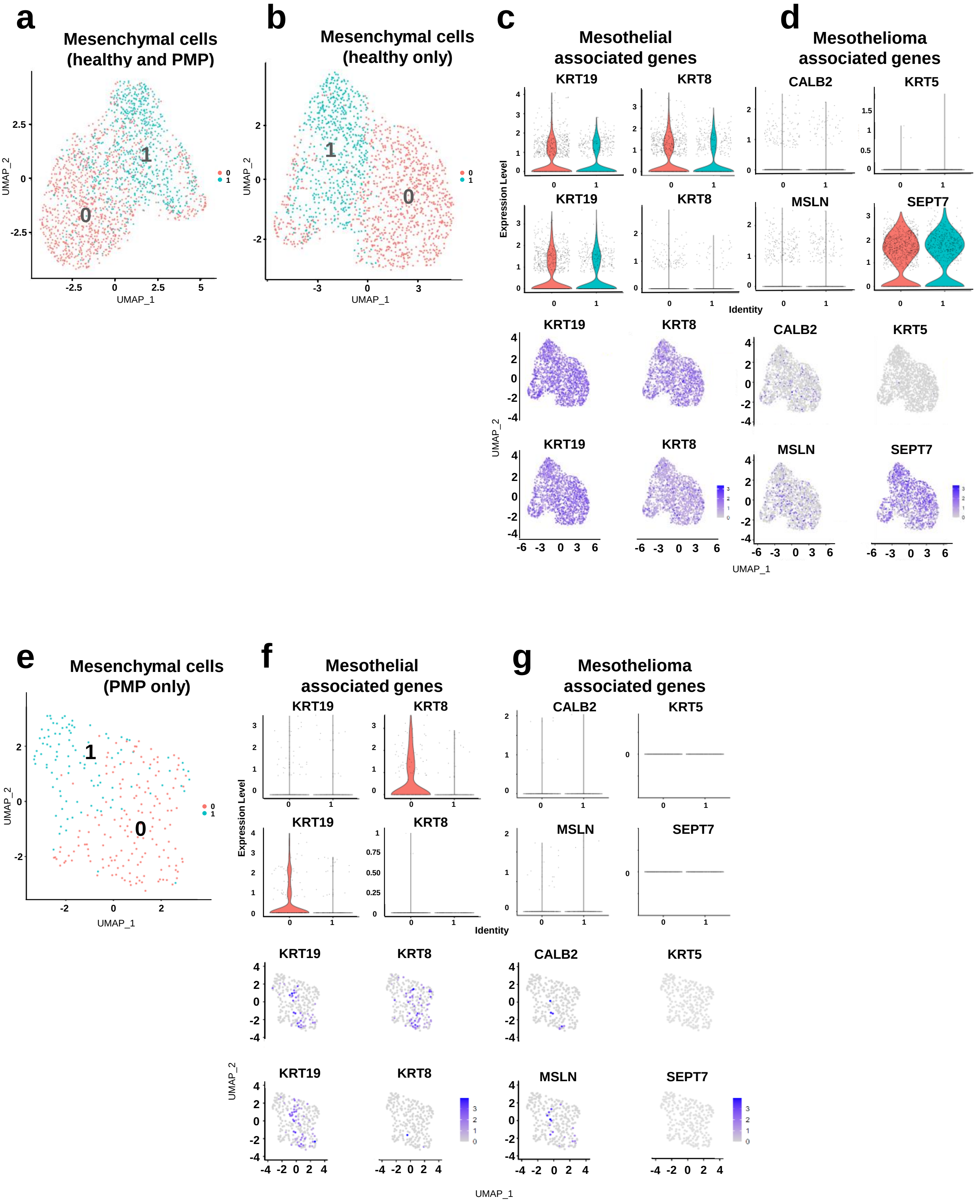

Fig. S11 Subset analysis of mesenchymal-characterized cells using uniform manifold approximation and projection (UMAP) plots.

(**a**) UMAP plot of mesenchymal cells, with colors representing Seurat-defined clusters. (**b**) UMAP plot of mesenchymal cells from healthy samples only, identifying two distinct Seurat clusters. (**c, d**) Expression patterns of (**c**) mesothelial (KRT family) markers and (**d**) mesothelioma markers, respectively, across Seurat clusters. (**e**) UMAP plot of mesenchymal cells from PMP samples only, identifying two distinct Seurat clusters. (**f, g**) Expression patterns of (**f**) mesothelial (KRT family) markers and (**g**) mesothelioma markers, respectively, across Seurat clusters.

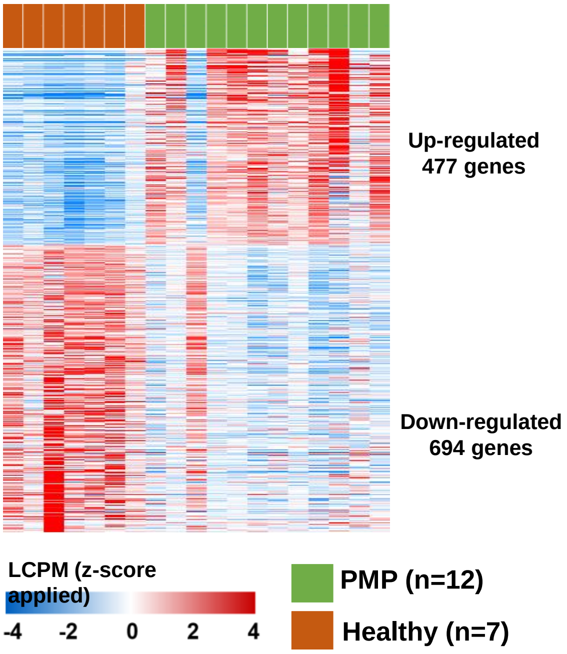

Fig. S12 Differentially expressed genes between healthy and pseudomyxoma peritonei (PMP) samples.

A total of 1,171 genes were significantly differentiated in expression between healthy sample and PMP subgroups in fresh frozen tissues (*P* < 0.001 and 1.5-fold difference or more in terms of expression levels). LCPM, log-transformed counts per million.

a

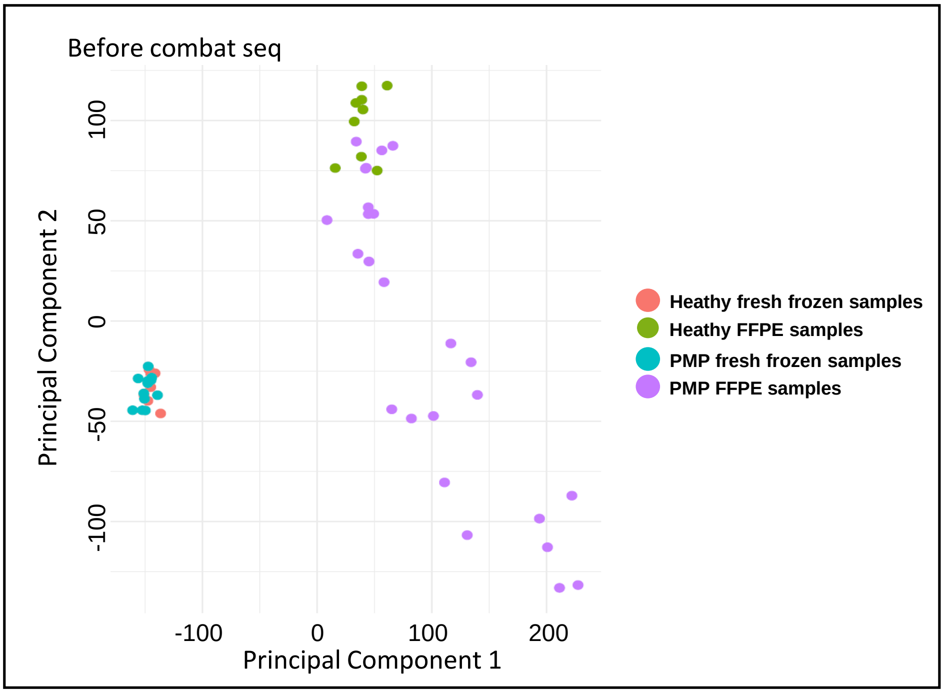

b

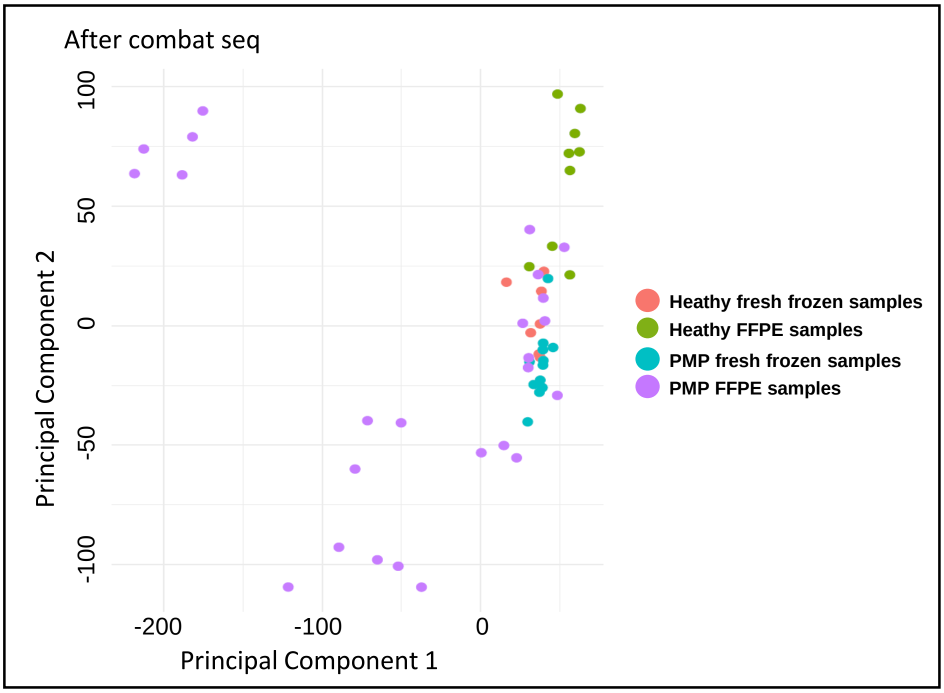

Fig. S13 Principal Component Analysis (PCA) before and after applying ComBat-seq for batch effect correction.

(**a**) PCA of fresh frozen tissue samples and formalin-fixed paraffin-embedded (FFPE) samples prior to ComBat-seq application. (**b**) PCA of fresh frozen tissue samples and FFPE samples following ComBat-seq application.

a

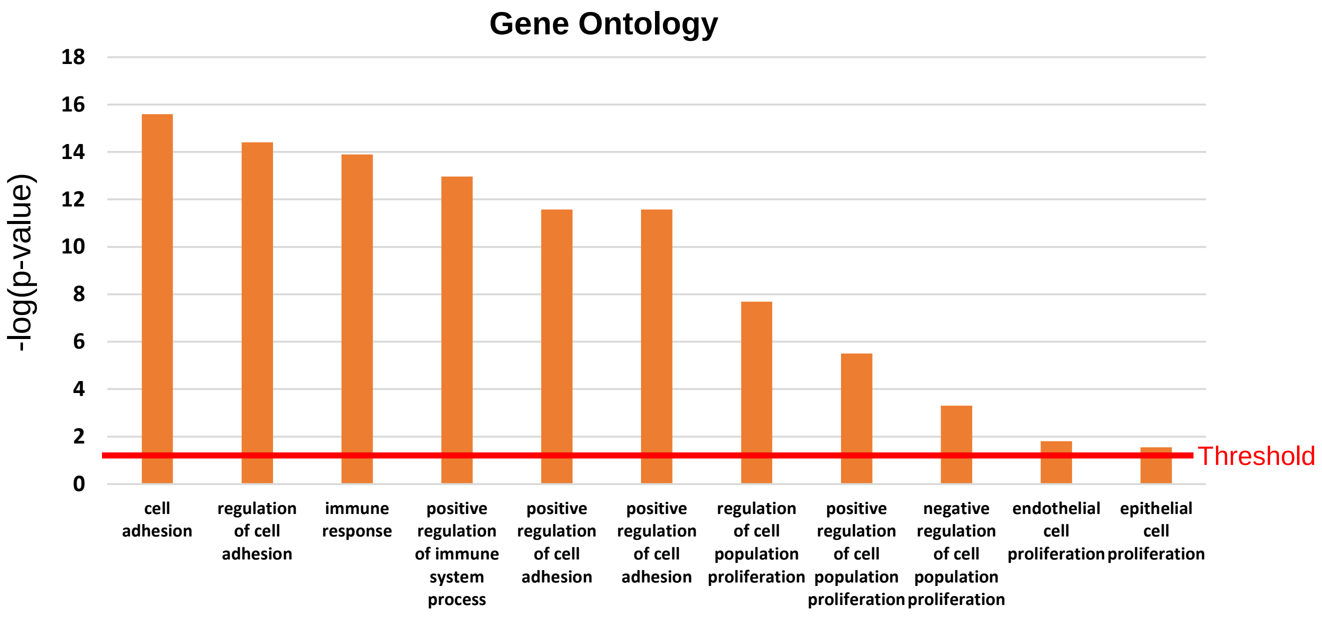

b

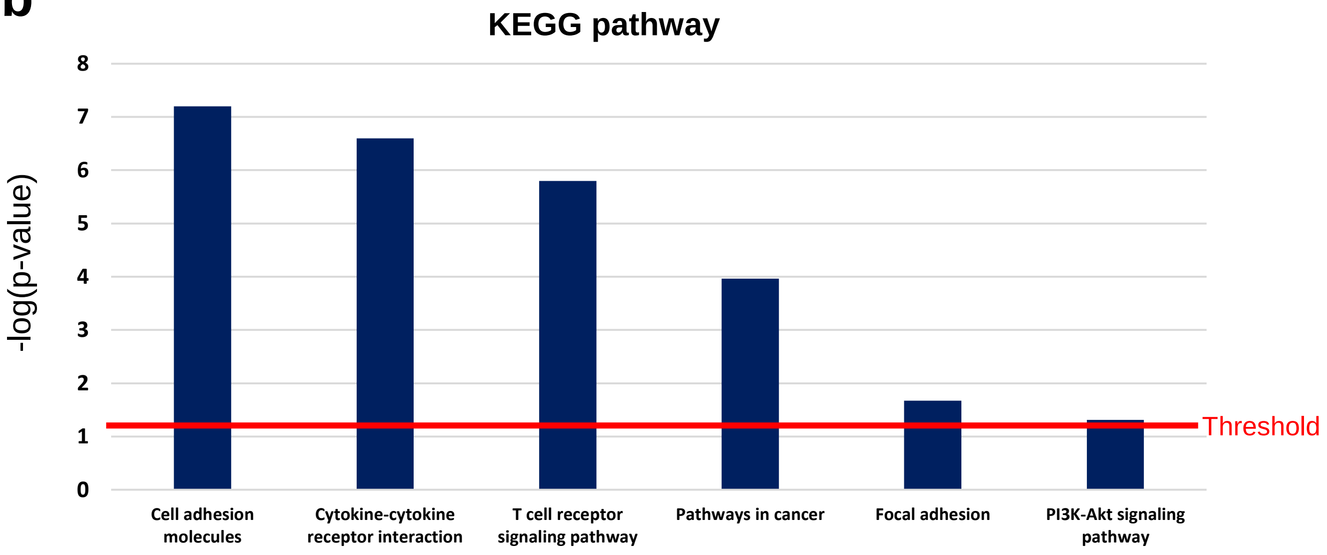

Fig. S14 Comparison of pseudo-bulk expression between pseudomyxoma peritonei (PMP) and healthy sample in single-cell functional enrichment analyses

Functional enrichment analysis of 1,967 differentially expressed genes between PMP and healthy sample using the pseudo-bulking technique. Enriched functions are shown for **(a)** Gene Ontology – Biological Process subcategory and **(b)** KEGG pathway.

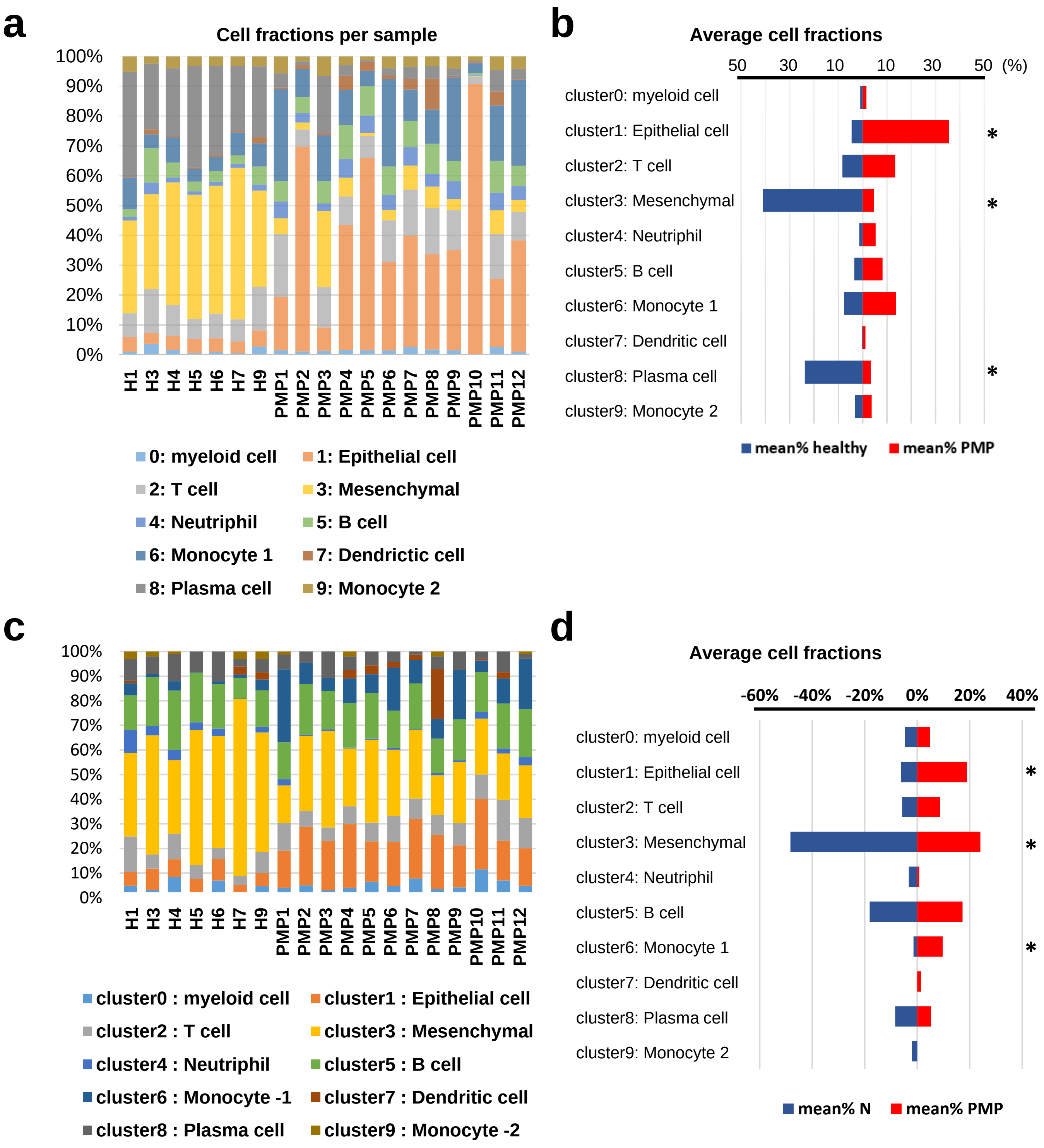

Fig. S15 Comparison of cell fractions between healthy (H) and pseudomyxoma peritonei (PMP) samples in bulk RNA-seq data.

(**a**) Cell fractions within cell groups for individual H and PMP samples analyzed using EPIC software. (**b**) Average cell fractions compared between H and PMP samples using EPIC. (**c**) Cell fractions within cell groups for individual H and PMP samples analyzed using CIBERSORTx. (**d**) Average cell fractions compared between H and PMP samples using CIBERSORTx. *$P < 0.05$, determined by Fisher's exact test.
